# Effects of Chronic Social Defeat Stress on Behavior and Dopamine Receptors in Adolescent Mice With 6-Hydroxydopamine Lesions of the Medial Prefrontal Cortex

**DOI:** 10.3389/fnbeh.2021.731373

**Published:** 2021-11-29

**Authors:** Tong Zhao, XiaoLei Gao, Guang-Biao Huang

**Affiliations:** ^1^Department of Psychiatry, The Second Affiliated Hospital of Xinxiang Medical University, Xinxiang, China; ^2^School of Nursing, Xinxiang Medical University, Xinxiang, China; ^3^Department of Psychiatry, Huzhou Third Municipal Hospital, The Affiliated Hospital of Huzhou University, Huzhou, China

**Keywords:** schizophrenia, social defeat stress, stress-susceptible, behavior, dopamine receptor, prefrontal cortex

## Abstract

**Background:** Social stress factors in schizophrenia have long-term effects, but will only induce symptoms in a portion of individuals, even if exposed to identical stress.

**Methods:** In the current experiment, we examined mice with 6-hydroxydopamine (6-OHDA)-induced medial prefrontal cortical (mPFC) injury to select for members of a “stress-susceptible group,” and observed the changes in their behavior and the expression of D1 and D2 dopamine receptors in the amygdala and hippocampus.

**Results:** We observed that after chronic social defeat stress, 72.6% of the 6-OHDA lesioned mice exhibited stress response to aggressors, compared to 52.3% of the blank control group. Both the 6-OHDA lesion + social defeat and social defeat groups exhibited anxiety and depression-like behavior. However, social cognitive impairment in the mice from the 6-OHDA lesion + social defeat group was more significant and the D1 expression levels in the amygdala were significantly decreased.

**Conclusion:** These results suggest that the reason that adolescent mice with cortical injury were highly sensitive to defeat stress and had more prominent social cognitive impairment may be the decreased selectivity of D1 in the amygdala.

## Introduction

A large body of research has amassed suggesting that social defeat stress research may be able to explain the role of environmental factors in the pathogenesis of schizophrenia. In clinical studies, migration, bullying, exclusion, and discrimination experiences are all risk factors for developing schizophrenia (Kelleher et al., 2008; Li et al., 2012; Selten et al., 2013). Social defeat stress and the aforementioned risk factors have similar face validity. Animal studies have reported an increased firing frequency of dopaminergic neurons in the ventral tegmental area, dopamine hypermetabolism in the marginal and cortical zones in the midbrain, and sensitization to amphetamines in social defeat animals (Tidey and Miczek, 1996; Yap et al., 2005; Cao et al., 2010; Razzoli et al., 2011). These results are similar to the pathophysiology of schizophrenia. This suggests that social defeat stress can be applied to schizophrenia etiology research (Selten and Cantor-Graae, 2008; Selten et al., 2013).

Krishnan et al. (2010) found that in mice with identical genetic backgrounds, only some will exhibit stress responses when exposed to social defeat. Meanwhile, it has been shown that repeated social defeat stress decreases stress-induced dopaminergic response in the medial prefrontal cortex (mPFC) and plays a critical role in stress susceptibility in rodents. Previous studies also revealed roles of mPFC projections to several brain areas, such as the basolateral amygdala (BLA), lateral habenula, and dorsal hippocampus, which have been suggested to be involved in stress responses (Brancato et al., 2014; Prabhu et al., 2018; Numa et al., 2019). Our team has also found that adolescent and adult mice can be divided into “stress-susceptible” and “stress-unsusceptible” groups after undergoing social defeat stress, with differences noted in anxiety and depression-like behaviors and cognitive function between the groups. In the “stress-susceptible” group, the expression of the endoplasmic reticulum stress proteins, namely, glucose-regulated protein 78 and CCAAT/enhancer-binding protein homologous protein in the amygdala was found to be significantly increased (Huang et al., 2013; Zhao et al., 2013). Yet, most of the previous social defeat stress research has failed to examine these differences in individual responses. Furthermore, cognitive dysfunction has been widely reported in patients with schizophrenia. Very little research has been conducted utilizing social defeat animals to report on this issue, with inconsistent results. For example, previous studies have reported the damage to the social and working memory of animals exposed to social defeat (Von Frijtag et al., 2000; Yu et al., 2011). However, our team members found that social defeat stress in the Morris water maze has no effects on learning ability in mice (Zhao et al., 2013).

Social stress factors in schizophrenia have long-term effects, but will only induce symptoms in a portion of individuals, even if exposed to identical stress. Therefore, the current project sought to extend the previous research on adolescent animals exposed to social defeat. Specifically, the current experiment examined mice with 6-hydroxydopamine (6-OHDA)-induced mPFC injury to select for members of a “stress-susceptible group,” and observed the changes in the behavior and the expression of D1 and D2 dopamine receptors in two brain regions: the amygdale and hippocampus.

## Materials and Methods

### Experimental Animals

Institute of Cancer Research-occluded mice, specifically, 30 days (12–14 g) male C57BL/6J mice (total of 80, *n* = 20 in each group) and 72 days (19–22 g) male mice (Beijing Vital River Laboratory Animal Technology Co., Ltd., China) at the time of delivery, were used throughout the study (Zhang et al., 2018). They were housed in groups (eight mice per cage) in a temperature-controlled room 22 ± 2°C, with 40–60% humidity, under a 12-h light/dark cycle (lights on for 07:00 h) with food and water *ad libitum* before the social defeat procedure. The mice were randomly divided into the following four groups: (1) control; (2) social defeat stress (SDS); (3) SDS + vehicle; (4) SDS + 6-OHDA. The randomized list was produced by staff members who were blinded to the study using Microsoft Excel (Microsoft Corporation, Washington, United States) according to a previous study (Jeehyoung and Wonshik, 2014). The blinded procedures were confirmed by the authorized agency of Xinxiang Medical University. The target number of subjects used in each experiment was determined based on the numbers in our previous studies. No statistical methods were used to predetermine the sample size. Dedicated efforts were made to minimize animal suffering and the number of animals used is in accordance with the Guidelines for Animal Experiments of the Xinxiang Medical University.

### Stereotaxic-Assisted Microinjection

**FIGURE 1:**
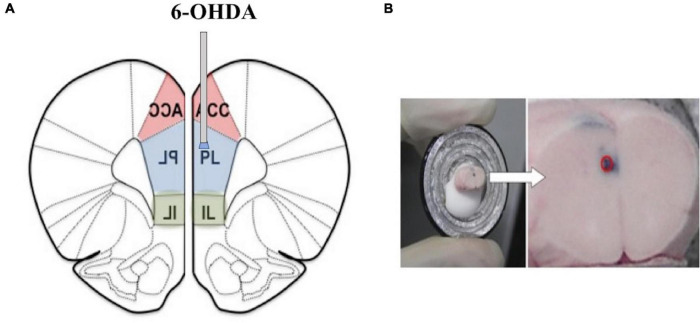
**(A)** A representative image of the locations of the tips of cannulas in the medial prefrontal cortex (mPFC) (1.8 mm anterior, 0.3 mm lateral, and 2.3 mm ventral from the bregma). **(B)** The red circle represents the injection.

Intraperitoneal injections of desipramine (25 mg/kg) and citalopram (20 mg/kg) were given to protect the adrenergic nerve fibers and serotonin nerve fibers 30 min before the 6-OHDA microinjections were administered to the animals (Tanaka et al., 2012). After the mice were anesthetized with isoflurane (Abbott Laboratories, Maidenhead, United Kingdom), they were fixed on the brain stereotaxic device. The microinjection device was inserted into the skull vertically, according to the confirmed coordinates, and the needle was slowly inserted to the predetermined depth: 1.8 mm anterior from the bregma, 0.3 mm lateral from the midline, and 2.3 mm ventral from the skull surface at the bregma according to a mouse brain atlas (Paxinos and Franklin, 2003) according to a previous study (Tanaka et al., 2012). The dopaminergic lesion in the mPFC was made as described previously (Roberts et al., 1994) with modifications. Before use, 6-OHDA was diluted to.5 mg/ml with the physiological saline containing.3 mg/ml ascorbic acid, and then 100 nl per injection site was pressure injected bilaterally in the mPFC regions using a PV-830 Pneumatic PicoPump (World Precision Instruments, Sarasota, FL, United States) through a glass micropipette made by a PN-30 micropipette puller (Narishige, Tokyo, Japan) and the needle was slowly withdrawn after 2 min (Figure 1). The SDS + vehicle group received the same treatment as the lesioned mice, except that saline instead of 6-OHDA was injected into the mPFC. Subsequent experiments were performed 7 days later.

### Social Defeat Stress Procedure

The social defeat stress procedure reported by Golden et al. (2011) was referenced in the current report. The C57BL/6J mice of the experimental group were placed as intruders in the selected Institute of Cancer Research (ICR) mouse cages for 10 min of physical contact. During the contact process, the C57BL/6J mice were attacked by the ICR mice, exhibiting evasion, compliance, resistance, and other behaviors. After 10 min of physical contact, a multi-pore transparent plastic board was used to separate the two mice, allowing the C57BL/6J mice to continuously receive psychological stress for the rest of the day (e.g., hearing sounds and/or smelling the odor of the ICR mouse). The C57BL/6J mice were rotated daily for 10 days to ensure that every mouse in the experimental group experienced stress from different aggressors.

### Behavioral Experiments

**FIGURE 2:**
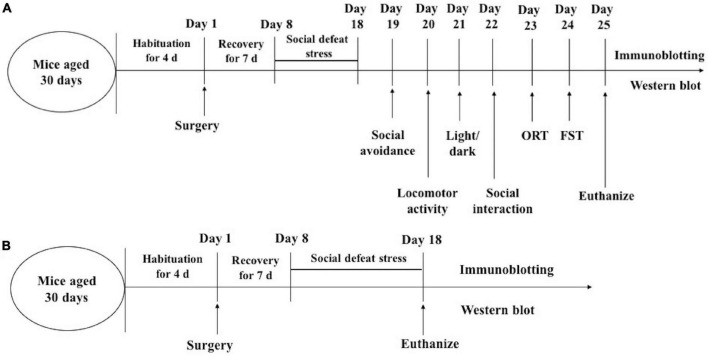
Timeline of experimental procedures. experiment 1 **(A)**; experiment 2 **(B)**. ORT, Object recognition test; FST, forced swimming.

In experiment 1, behavioral tests after the social defeat were carried out consecutively in the order of stress intensity between 09:00 and 16:00 h. After that, other behavioral tests were completed in sequence. One day after the behavioral tests (day 25), the mice were killed and the brain tissues were processed for Western blotting. In experiment 2, the mice were killed 1 day after the social avoidance tests (day 18) and the brain tissues were processed for Western blotting (Figure 2).

#### Social Avoidance Test

**FIGURE 3:**
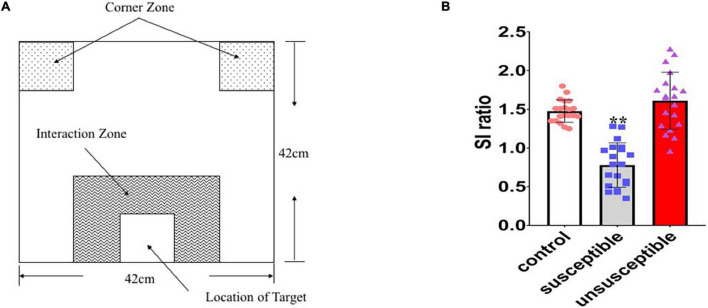
Identification of the susceptible and unsusceptible subgroups. **(A)** Schematic diagram of the social avoidance arena. **(B)** Susceptible mice (*n* = 20) had lower social interaction ratios than the control (*n* = 20) or resilient (*n* = 20) mice, indicating social avoidance behavior (F2,57 = 50.11, *p* < 0.0001).

Social avoidance testing was conducted in a box measuring 42 × 42 × 42 cm. The experiment was divided into two stages, with each stage taking up to 2.5 min. In the first stage, the test mice were placed at the far end of the interaction zone (defined as the 8-cm-wide area surrounding the wire mesh cage) and no ICR mouse was placed inside the interaction zone (Figure 3A). The mouse movement status in the open field was recorded. At the second stage, an ICR mouse (unfamiliar with the testing mouse) was placed in the small box inside the interaction zone and the test mouse was placed at the far end of the interaction zone at the same position. The interaction between the test mouse and the ICR mouse was observed. The entire experimental process used a video tracking system based on the spontaneous motor activity recording tracking (SMART) software (Smart software, Spain) for the recording and analysis to obtain an interaction ratio. The interaction ratio was calculated as 100 × (time spent in the interaction zone with an aggressor)/(time spent in the interaction zone without an aggressor). Based on previous works (Krishnan et al., 2007), an interaction ratio of 100 was set as a cut-off: mice with scores < 100 were defined as “susceptible.”

#### Locomotor Activity

The video tracking system with the SMART software was used for the automated recording of locomotor activity and was performed in an open acrylic box (30 × 40 × 50 cm). The mice were placed into the testing apparatus and their activity (distance moved) was immediately measured for 30 min. Subsequently, the time spent in the center (25%) and the area margins were determined.

#### Light/Dark Preference Test

The apparatus consisted of a rectangular acrylic box (46 × 27 × 30 cm), which included one large (27 × 27 cm) area and one small (18 × 27cm), separated by a door-like opening (7.5 × 7.5 cm) at the center. Each animal was individually placed at the center of the bright compartment (facing away from the door) and the following parameters were measured for 5 min: the total number of transitions between the light and dark areas; latency of the initial movement from the light to the dark area (latency of transition); total time spent in the light area.

#### Open Field Test

Test mice were placed in an open-field box measuring 50 × 40 × 30 cm. A video tracking system was used for the recording and analysis of the distance of movement and duration spent in the central region for 30 min.

#### Social Interaction Test

The test mice were placed in the aforementioned open-field box (30 × 40 × 50 cm) to observe their social interactions with the C57BL/6J mice from a different litter. A camera was used to record and analyze the social interaction behavior for 10 min, such as exploration of reproductive regions, social exploration, following, social grooming, as well as the duration and frequency of climbing.

#### Novel Object Recognition Test

The test mice were placed into the aforementioned open-field box for free exploration of 5 min to acclimatize to the environment, after which they were transferred to housing cages. After 5 min, the mice underwent training in the experiment box. At this time, there were two identical objects (sphere, cube, or triangular pyramid) which were arranged in a straight line 10 cm away from the box wall. The mice were placed from one of two corners and allowed to freely explore for 5 min before being transferred back to the housing cage. After a 5-min interval, the mice were placed in the experiment box for free exploration in the experiment period. During the novel object task, the time taken to explore the familiar object and the time taken to explore the novel object was recorded.

#### Forced Swim Test

The mice were placed in a glass cylindrical tank and their swimming activity was monitored for 6 min using a video camera. The total duration of immobility in the last 4 min was measured.

### Preparation of Brain Tissue

After the behavioral (day 25) and social defeat stress (day 18) tests were completed (Figure 2), the mice were terminated by decapitation under ether anesthesia. The amygdala and dentate gyrus regions of the dorsal hippocampus were punched out bilaterally using a 1-mm Harris Uni-Core micropunch (Electron Microscopy Sciences, Hatfield, Pennsylvania, United States).

### Immune Protein Blotting

Tissue samples were homogenized in 20 mM ice-cold Tris–HCl (pH 7.4) containing 1% protease and phosphatase inhibitor. The homogenates were centrifuged for 15 min at 4°C, and the resulting supernatant fractions were used for Western blot analyses. The membranes were blocked and incubated overnight at 4°C with a monoclonal mouse anti-D1R or polyclonal rabbit anti-D2R (Santa Cruz Biotechnology, Inc., Santa Cruz, California, United States) in 5% non-fat milk. On the next day, the membranes were washed with phosphate-buffered saline (PBS)-T and the primary antibody was detected using a goat anti-mouse horse-radish peroxidase (HRP)-linked immunoglobulin G (Santa Cruz Biotechnology Inc., Santa Cruz, California, United States) for D1, or goat anti-rabbit HRP-linked IgG (Vector Laboratories, Burlingame, California, United States) for D2 in PBS. The blots were developed using an enhanced chemiluminescence reagent (RPN2232; GE Healthcare, Inc., Piscataway, New Jersey, United States), visualized with a LAS-3000 Plus lumino-imaging analyzer (Fuji Photo Film Company, Kanagawa, Japan) and quantified using Multi Gauge software v3.0 (Fujifilm, Tokyo, Japan).

### Immunofluorescent Staining

The mice were perfused with ice-cold saline, followed by 4% (w/v) paraformaldehyde in PBS (pH 7.4). The brain tissue was fixed in 4% paraformaldehyde, dehydrated in graded ethanol, and embedded in paraffin. The coronal brain slices (30 μm or 10 μm thick) from the mPFC were sectioned with a frozen microtome (Leica, Wetzlar, Germany) to produce consecutive frozen sections. For the immunofluorescent staining, the sections were boiled in a citric acid buffer (pH 6) for 5 min in a microwave oven. After the sections were cooled, they were treated with 10% goat serum and 0.3% Triton X-100 for 1 h at room temperature. The sections were then incubated overnight at 4°C with a primary antibody (monoclonal rabbit anti-TH or anti-DβH antibody 1:200, Cell Signaling Technology, Inc., Danvers, Massachusetts, United States), the tissue sections were then washed in PBS (0.1 M, pH7.4, 3 × 5 min) and transferred for incubation with a secondary antibody [Goat anti-Mouse IgG (H + L) ThermoFisher Scientific, Inc., Waltham, Massachusetts, United States] at room temperature for 1 h. Finally, the slices were photographed by a fluorescent microscope (ECLIPSE-TC, Nikon Microsystems, Japan).

### Data Analysis

The data were analyzed with the GraphPad Prism version 8.0 software (Graphpad Software Inc., California, United States). The differences among the three groups were analyzed using one-way ANOVA. *Post hoc* individual comparisons were made using Tukey’s honestly significantly different test. The differences between the two groups were compared using *t*-tests. Spearman tests were used to assess relationships between various parameters in all mice. In all cases, differences were considered statistically significant at *p* < 0.05.

## Results

### Effects of the 6-Hydroxydopamine Lesioning of the Medial Prefrontal Cortex

**FIGURE 4:**
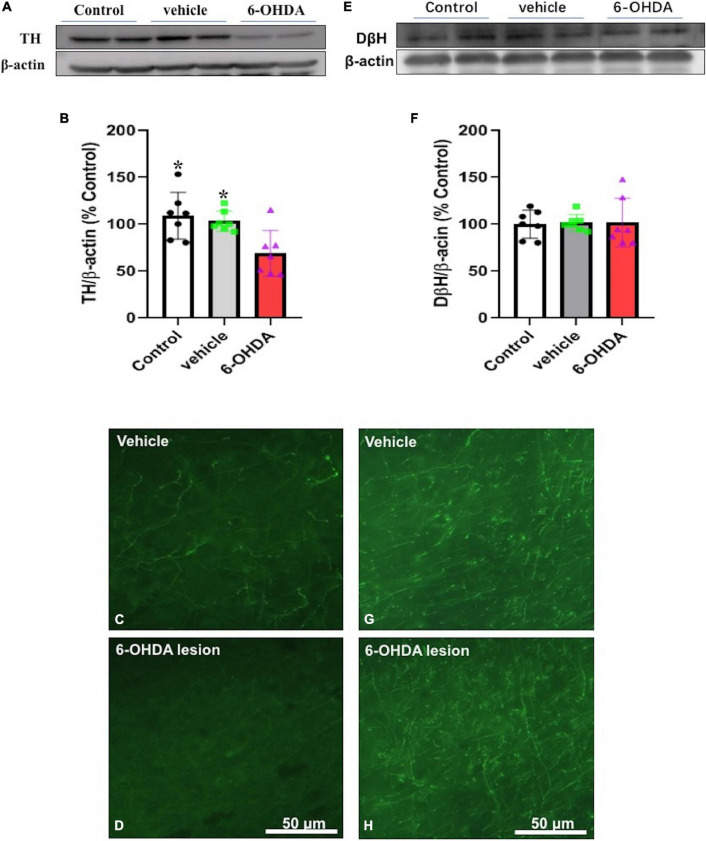
Immunoblotting or immunofluorescence was performed on Day 7 after the microinjection and protein expression of tyrosine hydroxylase **(A–D)** and dopamine beta-hydroxylase **(E–H)** in the mPFC of the vehicle control (vehicle) and the 6-hydroxydopamine (6-OHDA) microinjected mice * *p* < 0.05, compared with the 6-OHDA group (*n* = 7 per group).

Seven days following the 6-OHDA microinjection in the mPFC of the adolescent mice, the protein expression of tyrosine hydroxylase and dopamine beta-hydroxylase in the mPFC regions of the 6-OHDA treated group and the control group were quantitated. The results showed that the protein expression of tyrosine hydroxylase was significantly decreased after the 6-OHDA microinjection compared with the control group [*t* = 3.055 *p* = 0.01] and the vehicle group [*t* = 2.943 *p* = 0.0123] (Figures 4A–D) but no significant changes in the protein expression of dopamine beta-hydroxylase were observed (Figures 4E–H).

### Administration of Chronic Social Defeat Stress and Grouping

In the social defeat stress experiment, after all the ICR mice attacked the C57BL/6J mice (*n* = 200), all C57BL/6J mice were defeated and exhibited subordination (sideways or right submissive postures, immobility, fleeing, avoidance, lying on the back, or withdrawal), with 5.7% (*n* = 8) of the attacks resulting in fatality. The mice with injuries severe enough to affect the open-field test on Day 2 were excluded from further testing and analyses (*n* = 5).

The mice that underwent social defeat stress were subject to a final social avoidance test to screen stress-susceptible mice. The mice were placed into a box with ICR mice but were separated from direct contact with a metal mesh. The interactions between the test mice and the ICR mice were observed, and test mice with lower interaction rates (< 100) were designated the susceptible mice. Susceptible mice had lower social interaction ratios than unsusceptible mice [F2,57 = 50.11, *p* < 0.0001] (Figure 3B). After the chronic social defeat stress, 72.6% (*n* = 45) of the 6-OHDA lesioned mice (model group: 6-OHDA lesion + social defeat mice), 52.3% (*n* = 34) of the socially defeated mice (social defeat group), and 53.7% (*n* = 32) of the mice that received vehicle while suffering social defeat (social defeat + vehicle group) exhibited stress responses when exposed to the aggressor.

### Effect of Social Defeat Stress on Locomotion in the 6-Hydroxydopamine Lesioned Mice

**FIGURE 5:**
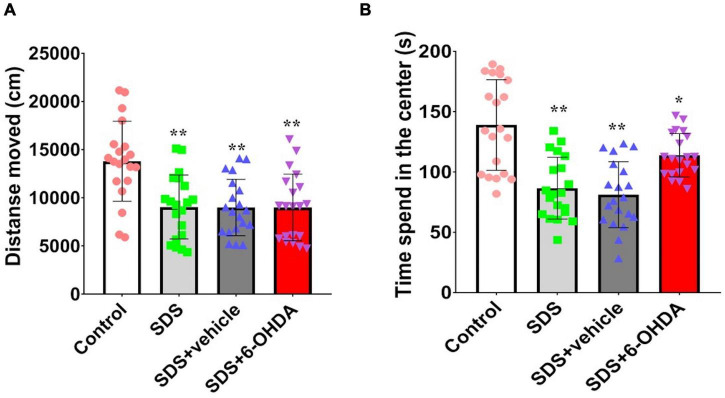
Effect of social defeat stress on locomotion in the 6-OHDA lesioned mice. **(A)**, Activity distance; **(B)**, Time of activity in the center. * *p* < 0.05, ** *p* < 0.01, compared with the control group (*n* = 20 per group).

There were statistically significant differences among the control group, social defeat group, and model group in terms of the time spent in the central region [*F*_(3_,_76)_ = 18.19, *p* < 0.0001] and movement distance [*F*_(3_,_76)_ = 9.359, *p* < 0.0001]. When the social defeat group was compared with the control group, the time spent in the central region [*t* = 5.155, *p* < 0.0001] and movement distance [*t* = 3.999, *p* = 0.0003] was significantly decreased. When the model group was compared with the control group, the time spent in the central region [*t* = 2.687, *p* = 0.0106] and movement distance [*t* = 3.966 *p* = 0.0003] was significantly decreased. However, there was no significant difference between the model group with the social defeat group in movement distance [*t* = 0.04008, *p* = 0.9682] and the time spent in the central [*t* = 2.013, *p* = 0.0513] (Figure 5).

### Effect of Social Defeat Stress on Light/Dark Preference in the 6-Hydroxydopamine Lesioned Mice

**FIGURE 6:**
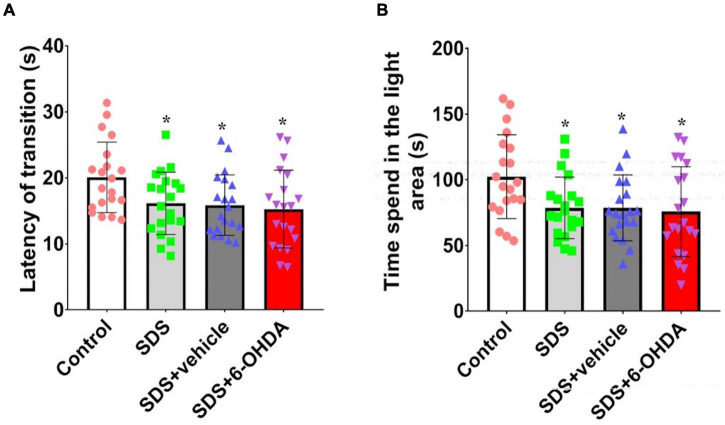
Effects of social defeat stress on light/dark preference in the 6-OHDA lesioned mice. **(A)**, Escape latency period; **(B)**, Time spent in the light area. * *p* < 0.05, compared with the control group (*n* = 20 per group).

There were statistically significant differences among the mice from the control group, social defeat group, and model group for the time spent in the light area [*F*_(3_,_76)_ = 3.679, *p* = 0.0157] and escape latency period [*F*_(3_,_76)_ = 3.587, *p* = 0.0175]. The social defeat group and the model group spent significantly less time in the light area [*t* = 2.680, *p* = 0.0108 or *t* = 2.552, *p* = 0.0148] and had significantly shorter escape latency periods [*t* = 2.469, *p* = 0.0181 or *t* = 2.698, *p* = 0.0104] than the control group. When the social defeat group was compared with the model group, the time in the light area [*t* = 0.3205, *p* = 0.7503] and escape latency period [*t* = 0.5090, *p* = 0.6137] (Figure 6).

### Effect of Social Defeat Stress on Social Interaction in the 6-Hydroxydopamine Lesioned Mice

**FIGURE 7:**
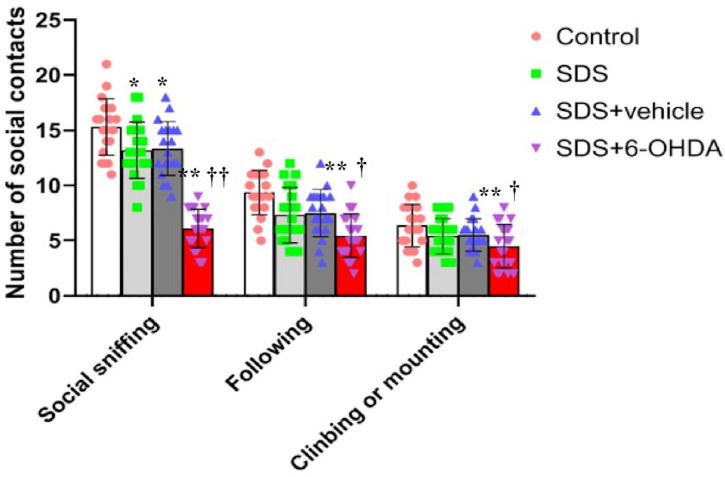
Effects of social defeat stress on the number of social interaction in the 6-OHDA lesioned mice. * *p* < 0.05, ** *p* < 0.01, compared with the control group; † *p* < 0.05, †† *p* < 0.01, when compared with the social defeat group (*n* = 20 per group).

When the control, social defeat, and model groups were compared, there were statistically significant differences in the frequency of social sniffing [*F*_(3_,_76)_ = 59.38, *p* < 0.0001], following [*F*_(3_,_76)_ = 6.433, *p* = 0.0006], and climbing/mounting [*F*_(3_,_76)_ = 4.479, *p* = 0.006]. When the mice in the model group were compared with the control and social defeat groups, the number of social sniffing [*t* = 13.29, *p* < 0.0001 or *t* = 10.29, *p* < 0.0001], following [*t* = 4.22, *p* = 0.0001 or *t* = 2.582, *p* = 0.0138], and climbing/mounting [*t* = 3.010, *p* = 0.0046 or *t* = 2.671, *p* = 0.0111] behaviors were decreased. When the mice in the social defeat groups were compared with the control group, a significant decrease in social sniffing was observed [*t* = 2.603, *p* = 0.0131]. However, there were no statistical differences in the number of following [*t* = 1.581, *p* = 0.1222] and climbing/mounting [*t* = 0.7452, *p* = 0.4608] behaviors (Figure 7).

### Effect of Social Defeat Stress on Novel Object Recognition in the 6-Hydroxydopamine Lesioned Mice

**FIGURE 8:**
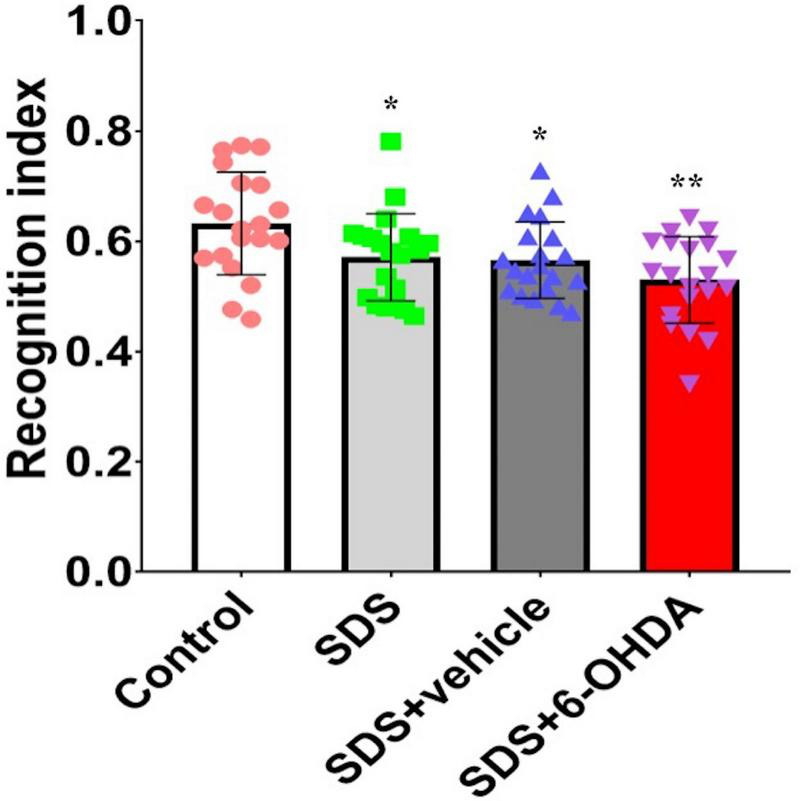
Effects of social defeat stress on the recognition index in the 6-OHDA lesioned mice. * *p* < 0.05, ** *p* < 0.01, compared with the control group (*n* = 20 per group).

Statistically significant differences in the recognition index among the control, social defeat, and model groups were noted [*F*_(3_,_76)_ = 5.596, *p* = 0.0016]. There were statistically significant differences in the recognition index between the social defeat and control groups [*t* = 2.242, *p* = 0.0309] and between the model and control groups [*t* = 3.768, *p* = 0.0006]. Although, there was no difference [*t* = 1.658, *p* = 0.1056] between the social defeat and model groups, when the model and social defeat groups were compared with the control group separately, the *p* level was different (Figure 8).

### Effect of Social Defeat Stress on Forced Swimming in the 6-Hydroxydopamine Lesioned Mice

**FIGURE 9:**
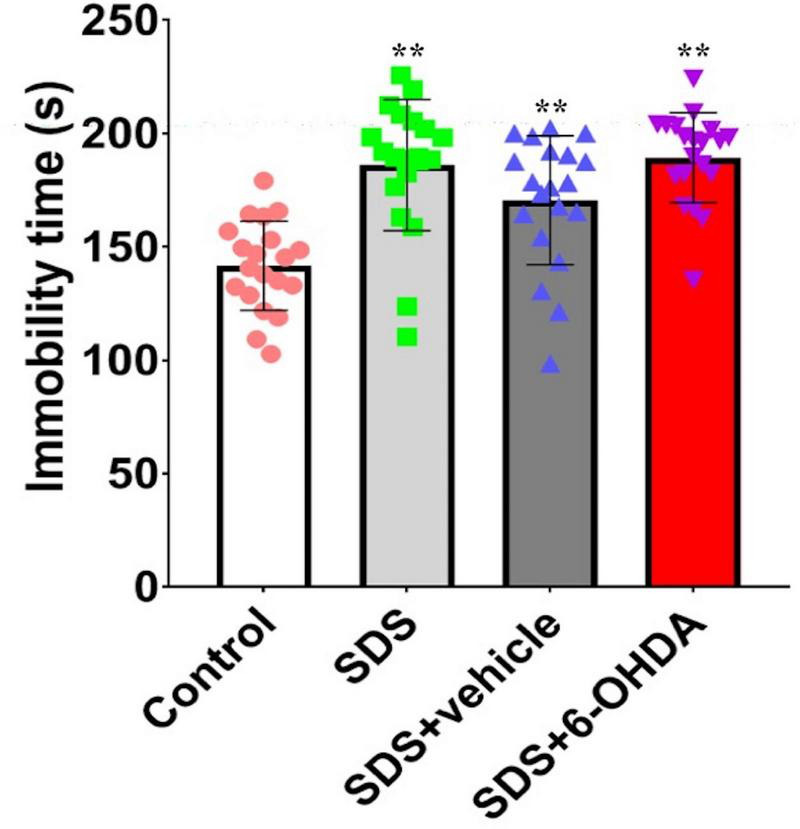
Effects of social defeat stress on forced swimming in the 6-OHDA lesioned mice. ** *p* < 0.01, compared with the control group (*n* = 20 per group).

The comparison of the three groups showed that there were statistically significant differences in the duration of immobility [*F*_(3_,_76)_ = 15.59, *p* < 0.0001]. Specifically, there were statistically significant differences in the duration of immobility between the social defeat and control groups [*t* = 5.668, *p* < 0.0001] and between the model and control groups [*t* = 7.629, *p* < 0.0001]. However, no difference [*t* = 0.4279, *p* = 0.6711] was noted between the social defeat and model groups (Figure 9).

### Effects of Social Defeat Stress on the Expression of D1 and D2 in the Amygdala and Hippocampus on the 6-Hydroxydopamine Lesioned Mice

**FIGURE 10:**
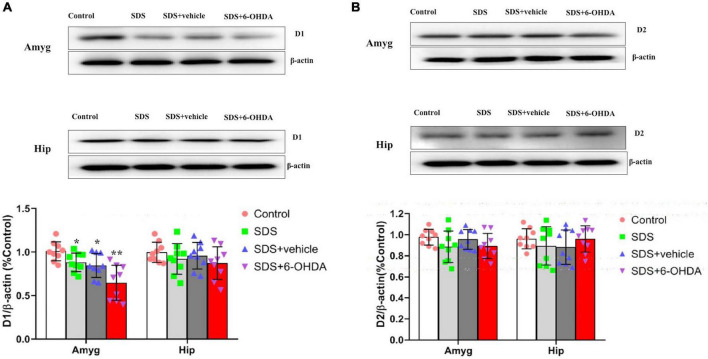
Effects of social defeat stress on the expression of D1 **(A)** and D2 **(B)** in the amygdala and hippocampus on the 6-OHDA lesioned mice. * *p* < 0.05, ** *p* < 0.01, compared with the control group (*n* = 9 per group).

There were statistically significant differences in the D1 expression in the amygdala between the control, social defeat, and model groups [*F*_(3_,_32)_ = 9.804, *p* < 0.0001]. There were statistically significant differences in the D1 expression levels in the amygdala when the control group was compared with the social defeat and model groups [*t* = 0.0211, *p* = 0.0211 or *t* = 4.742, *p* = 0.0002]. Although there was no statistical difference noted between the social defeat and model groups [*t* = 1.516, *p* = 0.149], when the model and social defeat groups were compared with the control group separately, the *p* level was different. When the hippocampal D1 expression levels were compared among the control, social defeat, and model groups, no statistical differences were noted [*F*_(3_,_32)_ = 0.9805, *p* = 0.4142] (Figure 10A).

There were no statistically significant differences in the D2 expression levels in the amygdala [*F*_(3_,_32)_ = 1.450, *p* = 0.2467] or hippocampus [*F*_(3_,_32)_ = 1.763, *p* = 0.5265] among the control, social defeat, and model groups (Figure 10B).

## Discussion

In this study, which was based on an animal model of neurodevelopmental disorders in schizophrenia, we examined the effects of social defeat stress on the behavioral changes and the dopamine receptors in adolescent mice with 6-OHDA-induced cortical injury. This study found that after chronic social defeat stress, 72.6% of mice in the model group exhibited stress response to aggressors, compared with 52.3% of the control group. The mice in the model and social defeat groups also exhibited anxiety and depression-like behavior. However, social cognitive impairment in the mice from the model group was more significant and the D1 expression levels in the amygdala were significantly decreased. The results suggest that the reason that the adolescent mice with cortical injury were highly sensitive to defeat stress and had more prominent social cognitive impairment may be the decreased selectivity of D1 in the amygdala.

Studies report that dopamine regulates the memory process at the cellular level through D1 receptors, as these receptors strengthen the function of neurons in the prefrontal cortex that are associated with memory (Arnsten et al., 1994). The 6-OHDA-induced dopamine deficiency in the prefrontal cortex causes the cognitive function to decline in mice (Slovin et al., 1999). Furthermore, the depression-like behavior and cognitive dysfunction caused by the chronic stress-induced decline in dopamine levels in mice can be reversed by selective agonists of dopamine D1 receptors (Rasheed et al., 2010). The blockade of dopamine D1-like receptor during social defeat restored social avoidance, furthermore, mPFC dopaminergic lesion by local injection of 6-OHDA facilitated induction of social avoidance upon social defeat (Tanaka et al., 2012). This shows that behavioral changes in mice with cortical injury after chronic stress may be associated with changes in the dopamine levels and abnormal expression of dopamine D1 receptors and partially supports our study findings. However, some studies show that dopamine D2 receptors play an important role in the memory process and some studies report that the use of D2 receptor agonists can improve memory efficiency (Luciana et al., 1998). Further, the cognitive function has shown a positive correlation with D2 receptor expression (Volkow et al., 1998). These results are not consistent with the conclusions of this study, signaling the need for further in-depth research to elucidate the specific mechanisms responsible.

Adolescence is a period where dopaminergic neurons develop and mature. Studies have shown that in rodents and monkeys, glutamatergic neurons and GABAergic neurons in the prefrontal cortex will develop and mature in the early developmental period following birth, while dopaminergic neurons develop and mature during adolescence (Spear, 2002). The onset of schizophrenia peaks during late adolescence and early adulthood, and dopamine in the prefrontal cortex is intimately associated with the cognitive disturbances observed in schizophrenia. Bergen and Coscia (Bergen and Coscia, 2001) reported that the brain development that occurs alongside the maturation of higher cognitive functions during adolescence will persist until early adulthood, and is affected by various factors, such as environment and experience, during this period (Toga et al., 2006). Because of this, we believe that developmental abnormalities in dopaminergic neurons during adolescence may damage social cognitive functions and are associated with the onset of schizophrenia. 6-hydroxydopamine has selective neurotoxic effects on dopaminergic neurons and associated fibers. The injection of 6-OHDA in the mPFC caused dopaminergic lesion, which is participated in social defeat stress. The effects of social defeat stress on dopaminergic nerve fibers in the adolescent mice with 6-OHDA-induced mPFC injury may be more significant than in adult mice. Previous studies have reported that under constant stress conditions, significantly increased c-Fos protein expression and significantly decreased social interaction behavior was observed in mice with a 6-OHDA-induced mPFC injury that occurs during adolescence when compared with injuries sustained in adulthood (Li et al., 2010; Sui et al., 2010).

The prefrontal cortex is an important region that is responsible for thinking, logical deduction, action planning, attention, working memory, higher cognitive function, and regulation of the stress response (Fuchs and Flügge, 2003; McDougall et al., 2004). In stress responses, mPFC pyramidal neurons send direct and indirect projections to various other brain areas, and meanwhile, mPFC dopaminergic activity appears to play a critical role in social avoidance. Research suggests that a decline in the dopamine function in the prefrontal cortex is closely linked with symptoms of schizophrenia and cognitive dysfunction. Animal model studies have found that both mice and primates with 6-OHDA induced prefrontal cortical injury demonstrate spatial memory impairment, decreased social interactions, and damage to cognitive functions (Bubser and Schmidt, 1990). There are reports that have suggested that social defeat animals have decreased dopamine levels in the mPFC, impaired social cognitive function, and reduced binding to dopamine D2 receptors in the basal ganglia (Brozoski et al., 1979; Bubser and Schmidt, 1990; Mitchell and Gratton, 1992; Taber et al., 1995; McDougall et al., 2004). When combined with the results of the current study, this suggests that after social defeat stress, the adolescent cortical injury animal model can better simulate cognitive dysfunction, which is one of the core symptoms of schizophrenia.

## Conclusion

In conclusion, this study is the first to report that mice with 6-OHDA induced cortical injury may be more suitable for association studies on social defeat stress and schizophrenia. This provides a scientific basis to explain the role of environmental factors in schizophrenia pathogenesis.

## Clinical Significance

Social stress factors in schizophrenia have long-term effects, but will only induce symptoms in a portion of individuals, even if exposed to identical stress. Therefore, selecting members of a “stress-susceptible group” is very important for early detection and treatment. In the current experiment, we examined mice with 6-OHDA-induced mPFC injury as the member of the “stress-susceptible group,” and observed the changes in the behavior and the expression of D1 and D2 dopamine receptors in various brain regions. In this study, we found that: (1)The adolescent mice with mPFC injury were highly sensitive to defeat stress. (2) Social cognitive impairment was more significant in the 6-OHDA lesioned mice. (3) The D1 expression levels significantly decreased in the amygdala from 6-OHDA lesioned mice. These results suggest that the reason that adolescent mice with cortical injury were highly sensitive to defeat stress and had more prominent social cognitive impairment may be the decreased selectivity of D1 in the amygdala. This provides a scientific basis for establishing a new generation of schizophrenia animal models to explain the role of environmental factors in schizophrenia pathogenesis.

## Data Availability Statement

The raw data supporting the conclusions of this article will be made available by the authors, without undue reservation.

## Ethics Statement

The animal study was reviewed and approved by The Second Affiliated Hospital of Xinxiang Medical University.

## Author Contributions

TZ and XG designed the study and wrote the protocol. G-BH commented on the protocol. XG undertook the statistical analysis. TZ, XG, and G-BH wrote the first draft of the manuscript. All the authors commented on the manuscript and contributed to and have approved the final manuscript.

## Conflict of Interest

The authors declare that the research was conducted in the absence of any commercial or financial relationships that could be construed as a potential conflict of interest.

## Publisher’s Note

All claims expressed in this article are solely those of the authors and do not necessarily represent those of their affiliated organizations, or those of the publisher, the editors and the reviewers. Any product that may be evaluated in this article, or claim that may be made by its manufacturer, is not guaranteed or endorsed by the publisher.
